# Unveiling the efficacy predictors and potential mechanisms of *Semen Cuscutae* against osteoporosis via machine learning and meta-analysis: a preclinical study

**DOI:** 10.3389/fimmu.2026.1766283

**Published:** 2026-06-10

**Authors:** Bo Dong, Rui Tang, Dongping Wan, Haoxiang Yuan, Chuan Leng, Rui Wang, Feilong Li, Junbo He, Yong Peng, Shihang Cao, Baohui Wang

**Affiliations:** 1 Department of Pain Management, Traditional Chinese Medicine Center, Honghui Hospital, Xi’an Jiaotong University, Xi’an, Shaanxi, China; 2 The Clinical Medical College, Chengdu University of Chinese Traditional Medicine, Chengdu, Sichuan, China; 3 The First Clinical Medical College, Guangxi University of Chinese Medicine, Nanning, Guangxi Zhuang Autonomous Region, China; 4 Department of Orthopedics and Traumatology, The Affiliated Traditional Chinese Medicine Hospital, Southwest Medical University, Luzhou, Sichuan, China

**Keywords:** machine learning, meta-analysis, osteoimmunology, osteoporosis, *Semen Cuscutae*

## Abstract

**Purpose:**

To systematically evaluate the anti-osteoporotic efficacy and underlying mechanisms of *Semen Cuscutae* and to explore potential experimental factors associated with treatment efficacy using an integration of meta-analysis and machine learning (ML).

**Methods:**

A comprehensive search of eight databases (from inception to December 1, 2025) was conducted to identify randomized controlled trials of *Semen Cuscutae* in osteoporotic animal models. Meta-analysis was performed to assess Bone Mineral Density (BMD), bone histomorphometry, and inflammatory/metabolic markers. Additionally, an exploratory XGBoost model interpreted via SHapley Additive exPlanations (SHAP) was constructed to quantitatively rank ten experimental variables potentially associated with therapeutic efficacy.

**Results:**

Fifteen studies were included in the final analysis. Meta-analysis demonstrated that *Semen Cuscutae* intervention significantly increased femoral and lumbar spine BMD, improved trabecular micro-architecture (increased BV/TV and Tb.Th; decreased Tb.Sp), and enhanced biomechanical strength. Mechanistically, Semen Cuscutae remodeled the osteoimmune microenvironment by suppressing pro-inflammatory cytokines (IL-6) and bone resorption markers (TRACP, CTX, RANKL), while ALP showed an increasing trend and osteogenic or reparative factors, including OPG, IGF, and TGF-β, were significantly upregulated. Exploratory ML analysis ranked “Species,” “Dose,” and “Treatment Duration” as the variables most strongly associated with therapeutic efficacy within the current dataset. Specifically, rat models exhibited superior therapeutic responsiveness compared to mice, and gender showed minimal predictive weight.

**Conclusion:**

*Semen Cuscutae* exerts robust osteoprotective effects via coupled anti-inflammatory and osteogenic mechanisms. The exploratory ML-derived ranking suggests that species-specific dose scaling and treatment duration may be important considerations for future preclinical and translational studies of *Semen Cuscutae*.

**Systematic Review Registration:**

https://www.crd.york.ac.uk/PROSPERO/, identifier CRD420261397983.

## 1 Introduction

Osteoporosis is a systemic skeletal disorder characterized by low bone mass and micro-architectural deterioration of bone tissue, leading to increased bone fragility and a consequent susceptibility to fracture (1; H. 2). It is clinically classified into two main categories: primary (e.g., postmenopausal and senile osteoporosis) and secondary (e.g., associated with endocrine disorders or medication use) (3). With the global aging population, the prevalence of osteoporosis is increasing. Particularly in China, Europe, and the United States, the incidence of osteoporotic fractures—such as hip and vertebral fractures—remains high, resulting in significant morbidity and mortality (4; L. 5). The pathophysiology primarily involves an imbalance in bone metabolism, specifically a disruption of the dynamic equilibrium between osteoblast-mediated bone formation and osteoclast-mediated bone resorption, which precipitates net bone loss (L.-T. 2). Underlying molecular mechanisms include the dysregulation of critical signaling pathways (such as the Wnt/β-catenin and RANKL/RANK/OPG axes), oxidative stress, chronic inflammation, and aberrant microRNA regulation (6). Furthermore, age, gender (postmenopausal status), genetics, nutritional status (deficiency in calcium and Vitamin D), lifestyle factors (smoking, sedentary behavior), and comorbidities (e.g., diabetes, hyperthyroidism) are established risk factors (6, 7). Clinically, fractures are associated with chronic pain, functional impairment, reduced quality of life, and a substantial long-term healthcare burden; notably, the one-year mortality rate following a hip fracture is as high as 20%–30% (8). Current therapeutic interventions have significant limitations. Bisphosphonates (e.g., alendronate) are associated with gastrointestinal adverse effects and a risk of medication-related osteonecrosis of the jaw (MRONJ) with long-term use (9, 10). Hormone replacement therapy has been linked to an increased risk of breast cancer and thromboembolic events (11). Although biologics such as denosumab (a RANKL inhibitor) are effective, they are costly and their discontinuation can lead to rebound bone loss. Moreover, poor adherence to complex regimens, such as those required for oral bisphosphonates, often compromises therapeutic efficacy (12, 13). Consequently, there is an urgent clinical need to develop novel therapies that are safer, more effective, and cost-efficient, particularly natural medicines with multi-target regulatory potential.

*Semen Cuscutae*, as a natural therapeutic agent, has garnered significant attention due to its remarkable osteoprotective properties (14). Modern pharmacological evidence indicates that *Semen Cuscutae* is rich in bioactive constituents, including flavonoids (e.g., hyperoside and kaempferol) and polysaccharides. These components have been shown not only to promote osteoblast differentiation and mineralization by activating critical signaling pathways such as Wnt/β-catenin and BMP-2, but also to significantly inhibit RANKL-induced osteoclastogenesis, thereby reversing bone loss (14–16). However, despite numerous independent animal studies reporting its anti-osteoporotic potential, current evidence remains fragmented, and there is a lack of rigorous quantitative meta-analyses to evaluate the overall effect size (17). More critically, existing research has predominantly focused on verifying single mechanisms. Consequently, key predictors of efficacy—such as optimal dosage, treatment duration, and variations in animal models—and the potential sources of heterogeneity remain unclear (16). This absence of evidence synthesis severely hinders the translation of *Semen Cuscutae* from basic research to clinical precision application.

While traditional meta-analysis remains the gold standard for evaluating intervention efficacy, it is often challenged by the high heterogeneity inherent in preclinical studies—such as variations in dosing protocols and animal species—and by complex, non-linear relationships (18). Recently, Machine Learning (ML) has emerged as a revolutionary tool for evidence synthesis. By leveraging its superior capabilities in high-dimensional data mining and pattern recognition, ML is progressively reshaping the research paradigm of evidence-based medicine (19–21). This study integrates meta-analysis with an exploratory ML-based feature-ranking approach. We aimed to quantitatively evaluate the anti-osteoporotic efficacy of *Semen Cuscutae* and to explore potential experimental factors associated with treatment efficacy through feature importance ranking. Importantly, the ML component was not intended to replace conventional meta-analytic approaches or to establish a confirmatory prediction model; rather, it was used as a complementary hypothesis-generating tool. Ultimately, this work seeks to provide preliminary evidence to support the translational evaluation of *Semen Cuscutae* in osteoporosis (22, 23; Y. 5).

## 2 Methods

This meta-analysis was conducted in strict accordance with the Preferred Reporting Items for Systematic Reviews and Meta-Analyses (PRISMA) guidelines (24) and was registered with PROSPERO (registration number: CRD420261397983).

### 2.1 Search strategy

A comprehensive literature search was performed across eight electronic databases, comprising Web of Science, PubMed, Embase, Scopus, the Foreign Medical Literature Retrieval Service (FMRS), China National Knowledge Infrastructure (CNKI), Wanfang Data, and the VIP Database. The retrieval process was conducted independently by two investigators. The search strategy utilized a combination of Medical Subject Headings (MeSH) terms and free-text keywords. The search covered the period from database inception to December 1, 2025. Detailed search strings and term combinations are provided in **Supplementary File 1**.

### 2.2 Inclusion and exclusion criteria

This study was designed as a systematic review of randomized controlled trials (RCTs) to evaluate the intervention efficacy of *Semen Cuscutae* extract compared with saline or placebo (vehicle control) in animal models of osteoporosis. The inclusion criteria were as follows: (a) Subjects: Successfully established animal models of osteoporosis; (b) Study type: *In vivo* experiments; (c) Outcomes: Clearly defined outcome measures with available extractable data; (d) Study design: Randomized controlled trials (RCTs). The exclusion criteria were: (a) Studies involving animal models with concurrent bone metabolic disorders (comorbidities); (b) *In vitro* experiments, or studies utilizing combination therapies or multi-herb formulations in the treatment group; (c) Duplicate publications or overlapping datasets; (d) Non-original research, including conference abstracts, literature reviews, editorials, and letters to the editor.

### 2.3 Data extraction

Following the removal of duplicates, two researchers independently screened the titles and abstracts of the remaining records in a blinded manner to exclude studies that did not meet the predefined criteria. Subsequently, the full-text articles of potentially eligible studies were reviewed to confirm adherence to the inclusion criteria. Any discrepancies arising during the screening process were resolved through discussion or by consultation with a third investigator to reach a consensus. Data extraction was performed independently by two investigators, strictly adhering to a blinded protocol. The following data were extracted: first author, year of publication, modeling method of osteoporosis, body weight and age of experimental animals, sample size, intervention regimen and administration route, duration of treatment (with specified time units), and the arithmetic mean and standard deviation (SD) of primary efficacy outcomes. For data presented exclusively in graphical format, numerical values were digitized and extracted using GetData Graph Digitizer software (Version 2.26).

### 2.4 Risk of bias assessment

The risk of bias of the included studies was independently assessed using the SYRCLE’s risk of bias tool (25). The assessment covered ten items across six domains: selection bias, performance bias, detection bias, attrition bias, reporting bias, and other sources of bias. Studies were classified as having a “low risk of bias” if all criteria were met, and a “high risk of bias” if criteria were not met. Items for which insufficient information was available to make a definitive judgment were categorized as “unclear risk of bias.” Any discrepancies between reviewers during the assessment process were resolved through discussion to ensure the accuracy and consistency of the results.

### 2.5 Outcome measures

The primary outcome measure was Bone Mineral Density (BMD), specifically assessed at the femur and lumbar spine. Secondary outcomes included: (1) bone histomorphometric parameters: trabecular number (Tb.N), trabecular thickness (Tb.Th), trabecular separation (Tb.Sp), and bone volume fraction (BV/TV); (2) bone biomechanical parameters: ultimate stress; (3) bone turnover markers: tartrate-resistant acid phosphatase (TRACP), C-terminal telopeptide of type I collagen (CTX), receptor activator of nuclear factor kappa-B ligand (RANKL), serum alkaline phosphatase (ALP), osteocalcin (OC), serum calcium (Ca), and serum phosphorus (P); and (4) cytokines: interleukin-6 (IL-6), osteoprotegerin (OPG), insulin-like growth factor (IGF), and transforming growth factor-beta (TGF-β).

### 2.6 Machine learning

To explore potential experimental factors associated with the anti-osteoporotic efficacy of *Semen Cuscutae*, a machine learning approach based on the gradient boosting framework was employed. Given the limited number of eligible studies, this analysis was designed as an exploratory and hypothesis-generating feature-ranking analysis rather than a confirmatory prediction model. Conventional multivariable meta-regression was not selected as the primary approach for evaluating all candidate moderators because the number of included studies was small relative to the number of prespecified study-level variables, which would make parameter estimation statistically unstable. Therefore, the ML analysis was used only as a complementary exploratory tool to generate a data-driven ranking of variables potentially associated with treatment efficacy. The results were not interpreted as causal effects, formal moderator tests, or externally validated predictions. First, a structured dataset comprising key experimental variables was constructed. The included features were: Species, Gender, Model type, Age, Weight, Active component, Route of administration, Vehicle, Dose, and Treatment duration. The target outcome for the ML analysis was the effect size of BMD improvement, expressed as BMD(SMD). Categorical variables were numerically encoded before model fitting, whereas continuous variables were retained in their original numerical form when available. Prior to modeling, Pearson correlation analysis was performed to generate a correlation heatmap, which was used as a preliminary visualization of pairwise associations among the encoded variables. Subsequently, the eXtreme Gradient Boosting (XGBoost) algorithm was applied to explore potential non-linear associations between experimental variables and treatment efficacy. The model was implemented using Python 3.13.5 with the xgboost package version 3.1.3 and scikit-learn version 1.8.0. A fixed random seed of 42 was used to improve reproducibility. The main model parameters were as follows: n_estimators = 50, max_depth = 2, learning_rate = 0.05, subsample = 0.8, colsample_bytree = 0.8, reg_lambda = 1.0, and objective = reg:squarederror. The 15 unique study-level records were randomly partitioned into a training set and a testing set at a ratio of 70:30. Model performance was summarized using R², root mean square error (RMSE), and mean absolute error (MAE). To address the “black-box” nature of machine learning models and quantify the contribution of specific factors, SHapley Additive exPlanations (SHAP) were introduced for model interpretability analysis. By calculating the mean absolute SHAP value for each feature, a global ranking of feature importance was established, thereby providing an exploratory ranking of variables potentially associated with the therapeutic efficacy of *Semen Cuscutae*.

### 2.7 Statistical analysis

Data processing, analysis, and visualization were performed using Stata SE (Version 18) and Review Manager (RevMan, Version 5.4.0). Heterogeneity was assessed using the I² statistic. A fixed-effects model was applied when the I² value was less than 50%. Conversely, when the I² value was ≥ 50%, a random-effects model (or fixed-effects model, contingent upon the identified source of heterogeneity) was selected. To further investigate potential sources of heterogeneity, subgroup analyses and sensitivity analyses were conducted. Sensitivity analysis was performed using the “leave-one-out” approach to validate the robustness of the results. For continuous outcomes, the choice of effect size was determined according to the consistency of measurement units and scales across studies. Mean difference (MD) with 95% confidence intervals (CIs) was used when outcomes were reported using the same unit and scale, whereas standardized mean difference (SMD) with 95% CIs was used when outcomes were measured using different units, scales, or measurement systems. The direction of the effect was defined according to the biological interpretation of each outcome. Positive effect sizes indicated higher values in the *Semen Cuscutae* group than in the control group, whereas negative effect sizes indicated lower values in the *Semen Cuscutae* group. Statistical significance was defined as p < 0.05; when the 95% CI included the null value, the result was interpreted cautiously as non-significant or as an increasing/decreasing trend when supported by the point estimate.

## 3 Results

### 3.1 Search results

**Figure 1:**
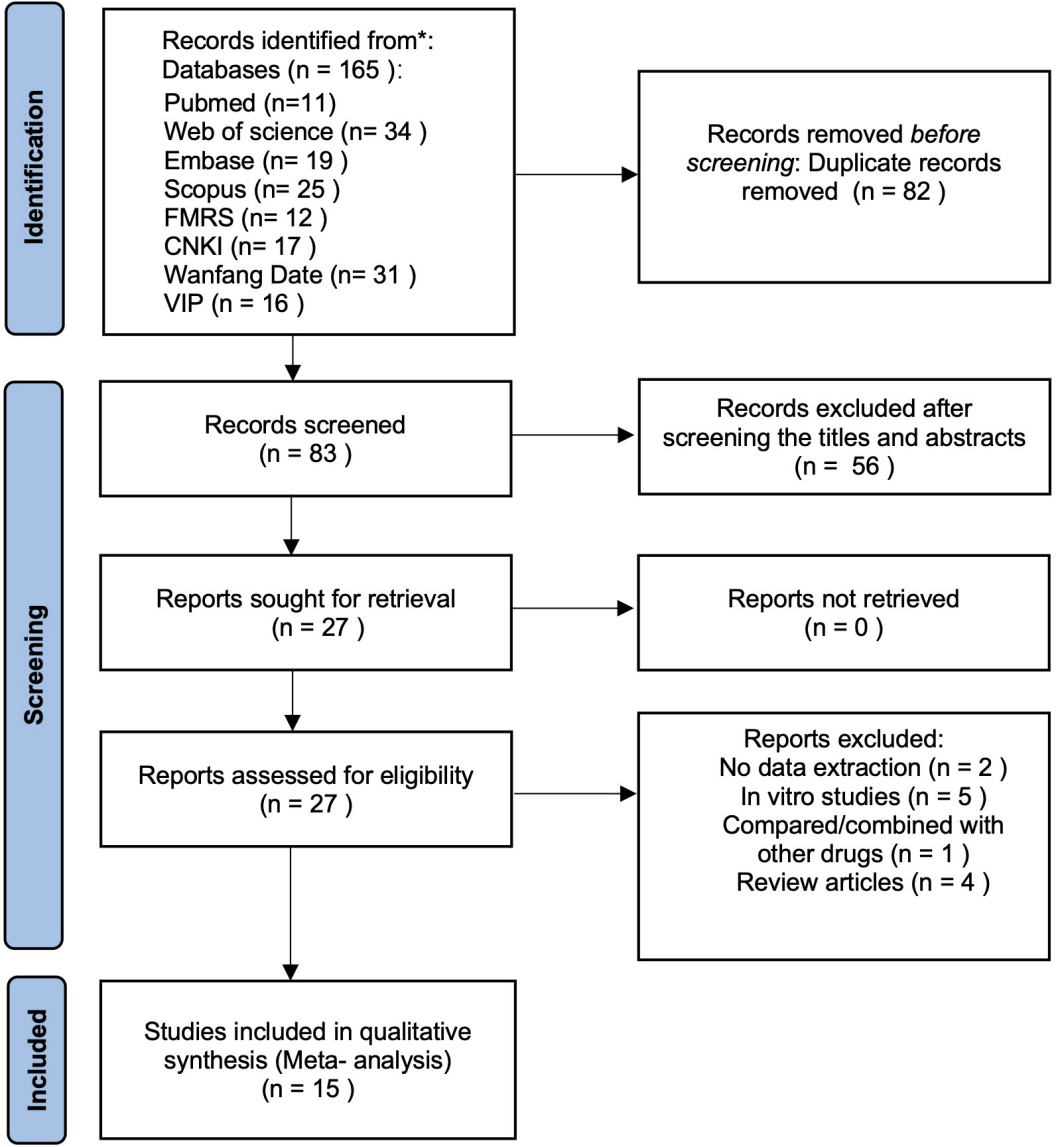
PRISMA flow chart of study selection.

The literature screening and selection process is illustrated in **Figure 1**. Initially, a total of 165 records were identified from eight Chinese and English databases. Following the removal of 82 duplicates, the titles and abstracts of the remaining records were screened, resulting in the exclusion of 56 irrelevant studies. The remaining 27 full-text articles were retrieved for detailed eligibility assessment. Subsequently, 12 studies were excluded for the following reasons: (a) unavailable data on primary outcomes (n = 2); (b) use of combined medication or comparison with other drugs (n = 1); (c) *in vitro* study design (n = 5); and (d) review articles (n = 4). Ultimately, 15 studies were included in the final analysis, comprising 9 Chinese articles (26–34) and 6 English articles (35–40).

### 3.2 Characteristics of included studies

The main characteristics of the included studies are summarized in **Table 1**. These studies were published between 2004 and 2025. Regarding the establishment of osteoporosis models, the majority (n = 13) utilized ovariectomy (OVX), while the remainder employed glucocorticoid induction (n = 1) or intragastric administration of retinoic acid (n = 1). In terms of animal species, 12 studies used rats, and 3 used mice. All studies specified the source of the active *Semen Cuscutae* components. Specifically, 8 studies utilized *Cuscuta chinensis* extract, and 4 used *Semen Cuscutae* flavonoids. The remaining 3 studies investigated specific active compounds: Matrine, Hyperoside, and Kaempferol, respectively. The routes of administration were intragastric gavage (n = 13) and injection (n = 2). Dosages ranged from 5 mg/kg/day to 5 g/kg/day, with intervention durations ranging from 4 to 12 weeks.

**Table 1:** characteristics of the included studies.

First author	Induction of osteoporosis	Species	Gender	Effective Substance	Sample size		Intervention		Methods of administration	Duration of study
					IG	CG	IG	CG		
(29)	Retinoic acid gavage	SD rat	Male	Cuscuta chinensis extract	8	8	200 mg/kg·d	PS	Intragastric	5 weeks
(30)	OVX	SD rat	Female	Cuscuta chinensis flavonoids	12	12	5 g/kg·d	PS	Intragastric	12 weeks
(27)	OVX	SD rat	Female	Cuscuta chinensis flavonoids	10	10	100 mg/kg·d	PS	Intragastric	12 weeks
(26)	OVX	SD rat	Female	Cuscuta chinensis flavonoids	11	11	3.6 g/kg·d	DW	Intragastric	12 weeks
(34)	OVX	SD rat	Female	Cuscuta chinensis flavonoids	20	20	180 mg/kg·d	DW	Intragastric	12 weeks
(33)	OVX	SD rat	Female	Matrine	6	7	33 mg/kg·d	PS	Intraperitoneal	8 weeks
(31)	OVX	SD rat	Female	Cuscuta chinensis extract	16	15	20 μg/ml·d	PS	Subcutaneous	10 weeks
(32)	OVX	SD rat	Female	Cuscuta chinensis extract	10	10	0.7 g/kg·d	PS	Intragastric	12 weeks
(28)	OVX	C57BL/6J mouse	Female	Cuscuta chinensis extract	10	10	4 g/kg·d	PS	Intragastric	12 weeks
(40)	OVX	C57BL/6J mouse	Female	Cuscuta chinensis extract	20	20	4 g/kg·d	PS	Intragastric	8 weeks
(37)	Hormone induction	SD rat	Female	Cuscuta chinensis extract	8	8	100 mg/kg·d	PS	Intragastric	4 weeks
(39)	OVX	C57BL/6J mouse	Female	Hyperoside	8	8	80 mg/kg·d	PS	Intragastric	10 weeks
(38)	OVX	Wistar rat	Female	Kaempferol	8	8	5 mg/kg·d	PS	Intragastric	8 weeks
(35)	OVX	SD rat	Female	Cuscuta chinensis extract	8	8	1.08 g/kg·d	PS	Intragastric	12 weeks
(36)	OVX	Wistar rat	Female	Cuscuta chinensis extract	8	8	400 mg/kg·d	PS	Intragastric	12 weeks

OVX, Ovariectomy; IG, Intervention Group; CG, Control Group; PS, physiological saline; DW, Distilled water.

### 3.3 Quality assessment of included studies

**Figure 2:**
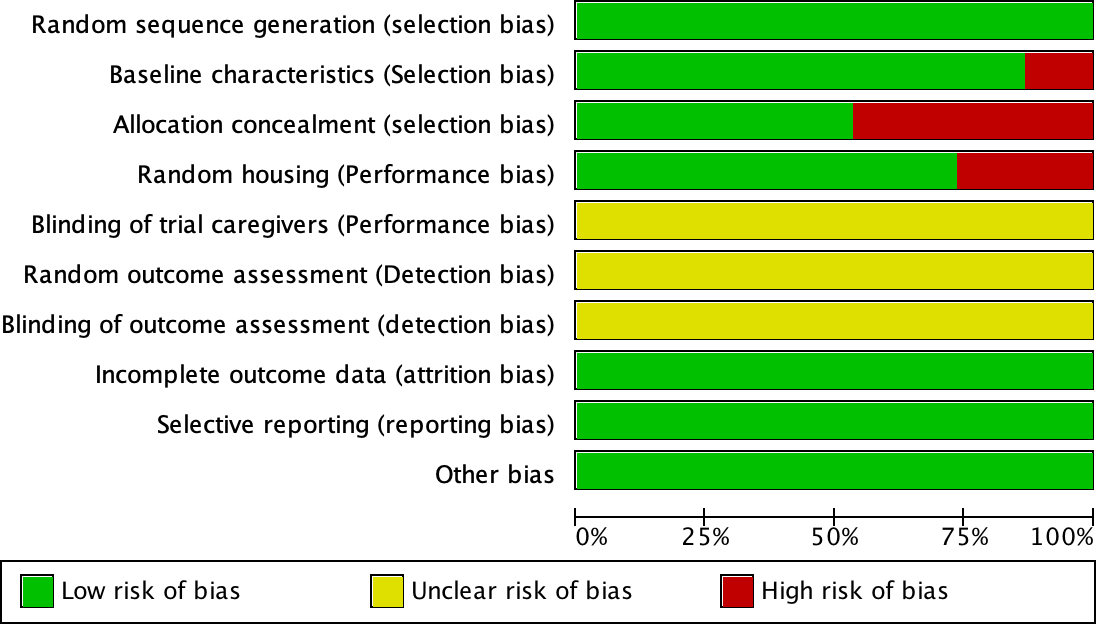
Quality assessment of the included studies.

The risk of bias assessment using the SYRCLE tool is summarized in **Figure 2**. All included studies reported adequate sequence generation and were free from risks related to incomplete outcome data, selective reporting, and other sources of bias. However, incomplete reporting of baseline characteristics was noted in 2 studies, and allocation concealment was not reported in 7 studies. Additionally, 4 studies did not clearly specify random housing conditions. Notably, none of the included studies reported blinding of caregivers, random outcome assessment, or blinding of outcome assessors. These reporting deficiencies indicate potential risks of performance and detection bias, which should be considered when interpreting the pooled estimates.

### 3.4 BMD and subgroup analysis

**Figure 3:**
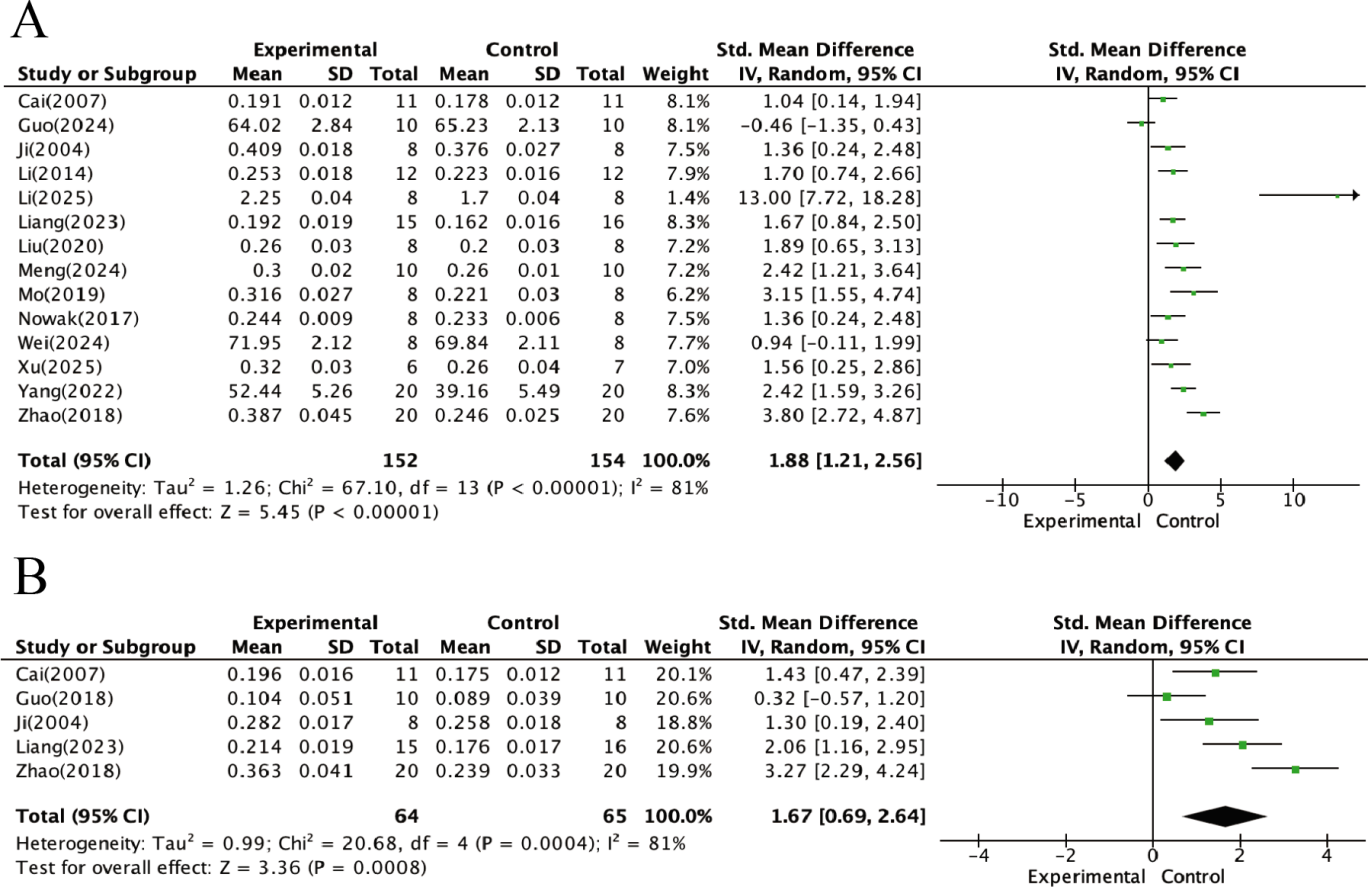
Forest plot comparing BMD between the *Semen Cuscutae* group and the control group. **(A)** Femur BMD; **(B)** Lumbar spine BMD. Effect size: SMD. Positive values indicate higher BMD in the *Semen Cuscutae* group and therefore favor *Semen Cuscutae*.

The meta-analysis of data from 15 studies confirmed the efficacy of *Semen Cuscutae* in improving Bone Mineral Density (BMD) in animal models of osteoporosis (**Figure 3**). As shown in **Figure 3A**, femur BMD in the *Semen Cuscutae* treatment group was significantly higher than that in the control group (SMD = 1.88, 95% CI: 1.21–2.56, *p* < 0.001). Similar improvements were observed in lumbar spine BMD (**Figure 3B**; SMD = 1.67, 95% CI: 0.69–2.64, *p* < 0.001). To investigate potential sources of heterogeneity in femur BMD, subgroup analyses were conducted based on species, active component, dosage, and duration of intervention (**Table 2**). A substantial reduction in I² values was observed in subgroups stratified by dosage and intervention regimen, suggesting that these factors may be the primary sources of heterogeneity. The results indicated that *Semen Cuscutae* intervention exhibited superior efficacy in rats, at medium dosages (100–1000 mg/kg/day), and with longer intervention durations (> 10 weeks). Conversely, no statistically significant difference was observed in the mouse models, suggesting that further large-scale studies are warranted to verify these findings in mice.

**Table 2:** Subgroup analysis of BMD based on multiple factors related to Semen Cuscutae treatment.

Subgroup	Standardized mean difference (95% confidence interval)	I^2^	p value
Species			
Rat	2.10 [1.38, 2.82]	75	0.000
Mice	0.97 [-0.77, 2.72]	91	0.27
Effective Substance			
Cuscuta chinensis extract	2.12 [1.03, 3.20]	85	0.000
Cuscuta chinensis flavonoids	2.16 [0.59, 3.72]	87	0.000
Other	1.22 [0.56, 1.88]	0	0.000
Dose			
≤ 100 mg/(kg·d)	1.60 [1.02, 2.18]	25	0.000
- 1000 mg/(kg·d)	2.38 [1.30, 3.46]	71	0.000
> 1000 mg/(kg·d)	1.97 [0.38, 3.55]	90	0.000
Duration of intervention			
≤ 10 weeks	1.73 [1.24, 2.22]	31	0.000
> 10 weeks	2.27 [0.89, 3.64]	90	0.000

### 3.5 Bone histomorphometry and biomechanical parameters

**Figure 4:**
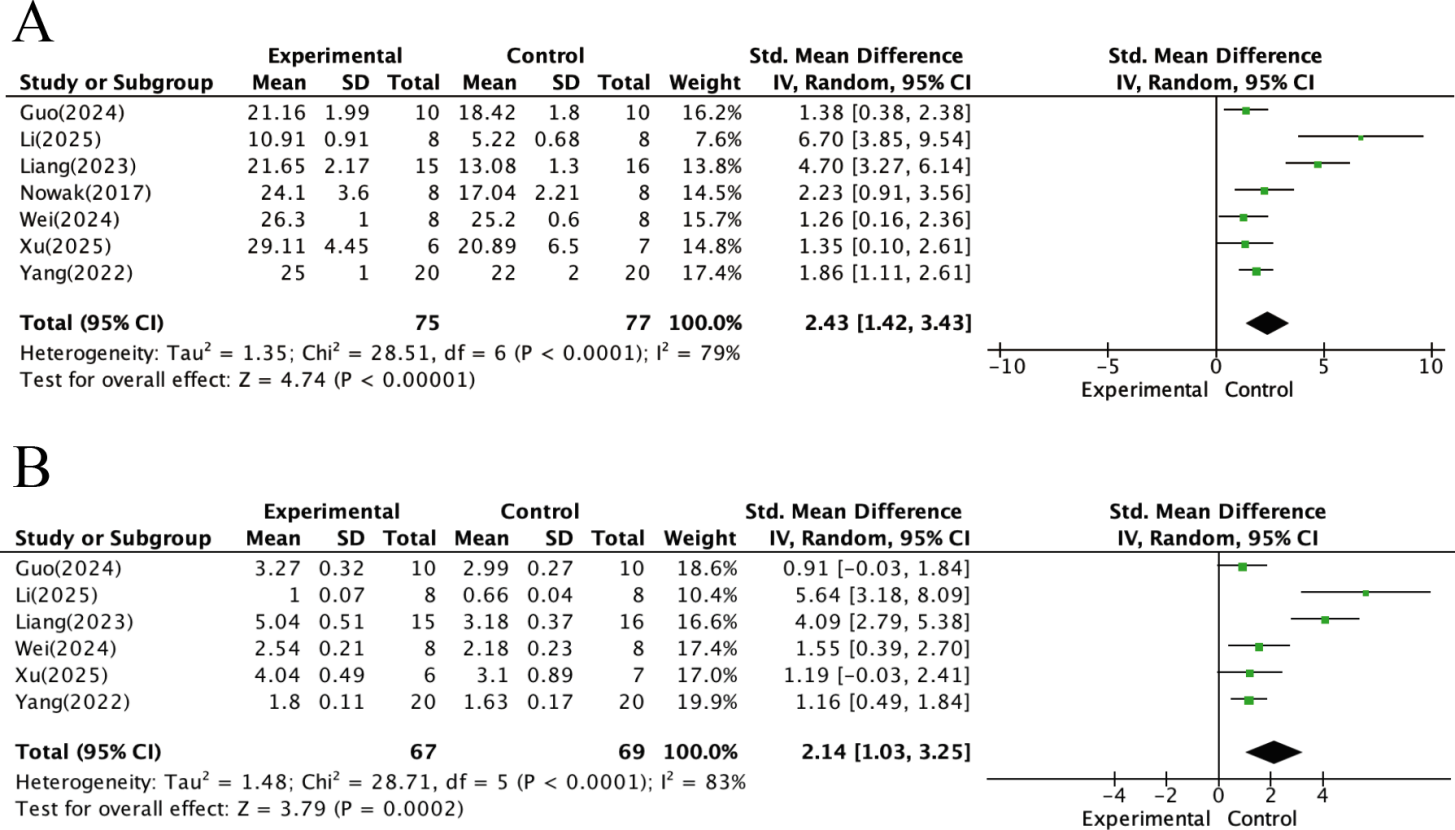
Forest plot of bone histomorphometric parameters. **(A)** Bone volume fraction (BV/TV); **(B)** Trabecular number (Tb.N). Effect size: SMD. Positive values indicate higher BV/TV or Tb.N in the *Semen Cuscutae* group and therefore favor *Semen Cuscutae*.

**Figure 5:**
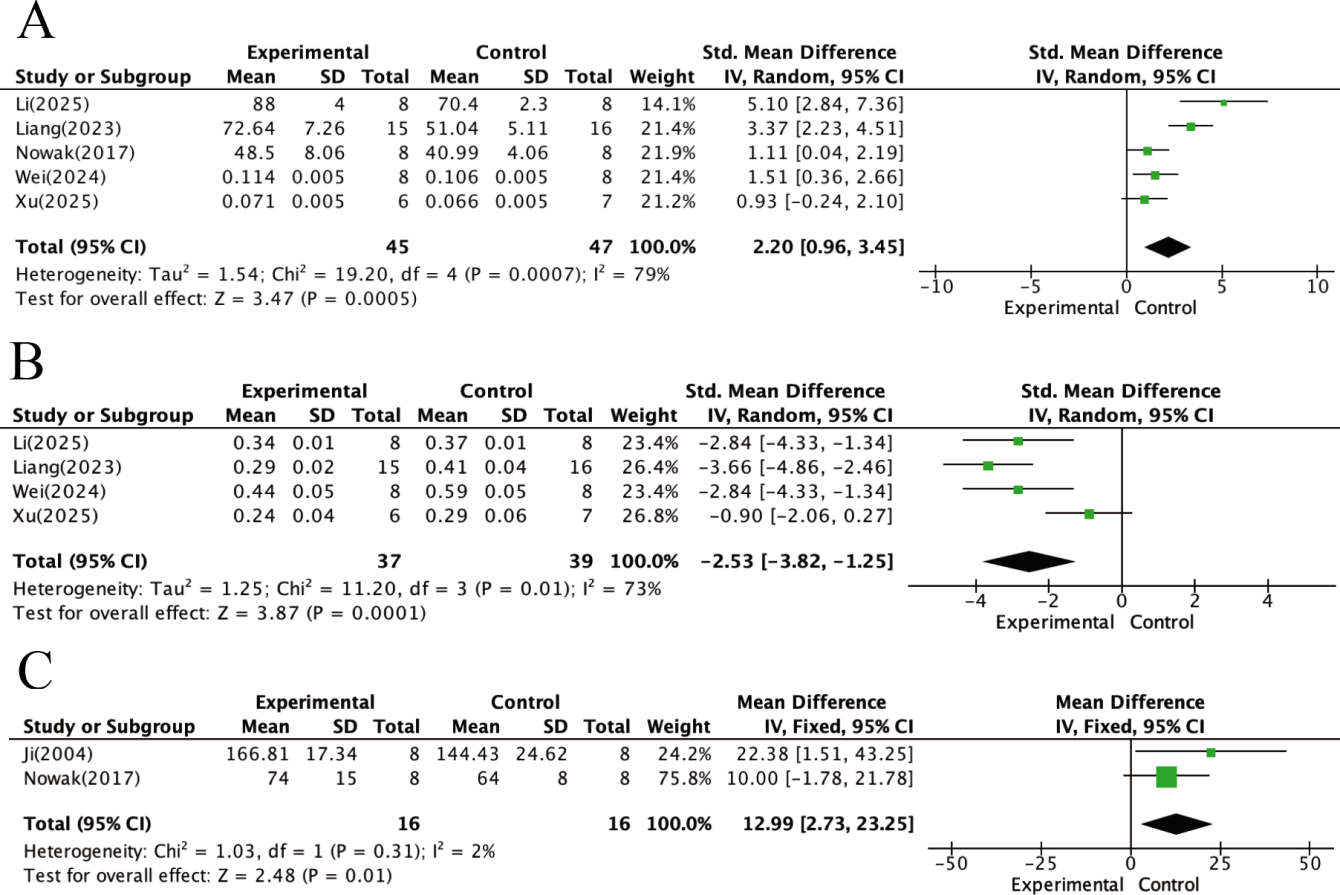
Forest plot of bone histomorphometric and biomechanical parameters. **(A)** Trabecular thickness (Tb.Th); **(B)** Trabecular separation (Tb.Sp); **(C)** Ultimate stress. Effect size: SMD for Tb.Th and Tb.Sp, and MD for ultimate stress. Positive values for Tb.Th and ultimate stress indicate higher values in the *Semen Cuscutae* group and favor *Semen Cuscutae*; negative values for Tb.Sp indicate lower trabecular separation in the *Semen Cuscutae* group and favor *Semen Cuscutae*.

**Figures 4**, **5** illustrate the effects of *Semen Cuscutae* intervention on bone histomorphometry and biomechanical parameters in osteoporotic animal models. As shown in **Figure 4**, seven studies reported significant improvements in bone volume fraction (BV/TV) (SMD = 2.43, 95% CI: 1.42–3.43, *p* < 0.001). Additionally, data from six studies demonstrated a positive effect of *Semen Cuscutae* on trabecular number (Tb.N) (SMD = 2.14, 95% CI: 1.03–3.25, *p* < 0.001). **Figure 5** summarizes the findings regarding trabecular thickness (Tb.Th), trabecular separation (Tb.Sp), and ultimate stress. Five studies indicated that *Semen Cuscutae* significantly increased Tb.Th (SMD = 2.20, 95% CI: 0.96–3.45, *p* < 0.001). Conversely, four studies showed a significant reduction in Tb.Sp (SMD = -2.53, 95% CI: -3.82 to -1.25, *p* < 0.001). Furthermore, two studies analyzed ultimate stress, showing a significant increase (MD = 12.99, 95% CI: 2.73–23.25, *p* = 0.01).

### 3.6 Bone metabolic indices

**Figure 6:**
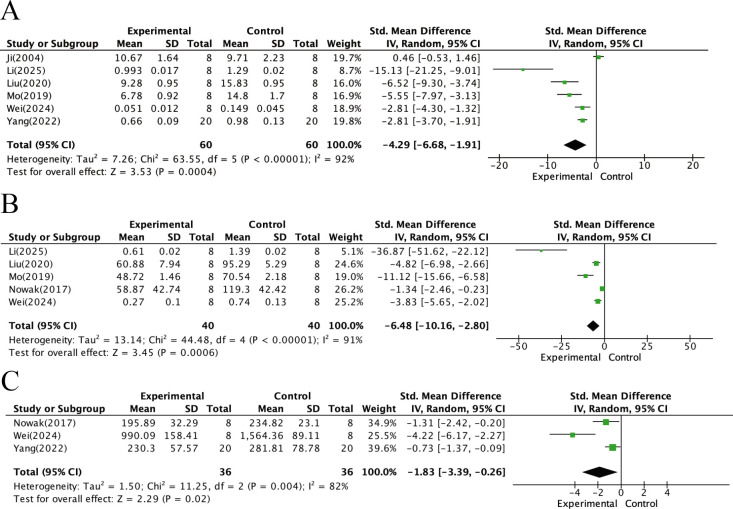
Forest plot of bone resorption markers. **(A)** TRACP; **(B)** CTX; **(C)** RANKL. Effect size: SMD. Negative values indicate lower levels of bone resorption markers in the *Semen Cuscutae* group and therefore favor *Semen Cuscutae*.

**Figure 7:**
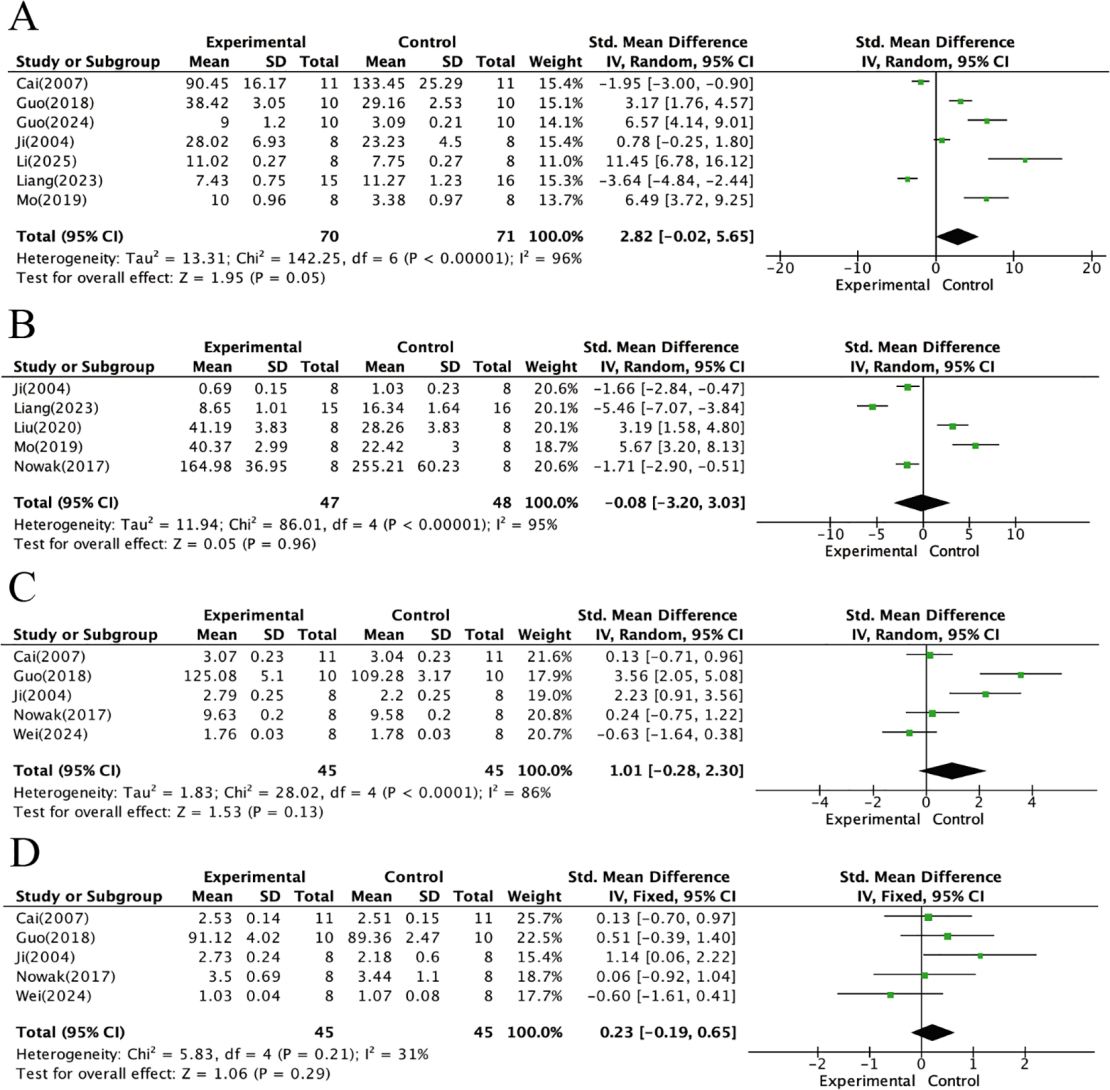
Forest plot of bone formation and biochemical markers. **(A)** ALP; **(B)** OC; **(C)** Serum calcium (Ca); **(D)** Serum phosphorus (P). Effect size: SMD. Positive values indicate higher levels in the *Semen Cuscutae* group, whereas negative values indicate lower levels. The ALP result was interpreted as an increasing trend because its 95% CI crossed zero.

**Figures 6**, **7** present the meta-analysis results regarding the effects of *Semen Cuscutae* on bone metabolism and biochemical markers in osteoporotic animal models. As shown in **Figure 6**, nine studies reported on TRACP levels, indicating a significant reduction in the treatment group (SMD = -4.29, 95% CI: -6.68 to -1.91, *p* < 0.001). Similarly, five studies summarized the results for CTX, showing a significant decrease (SMD = -6.48, 95% CI: -10.16 to -2.80, *p* < 0.001). Three studies analyzed RANKL levels, also demonstrating a significant reduction (SMD = -1.83, 95% CI: -3.39 to -0.26, *p* = 0.02). **Figure 7** summarizes the findings for other biochemical markers. Seven studies reported on ALP, which showed an increasing trend in the *Semen Cuscutae* group (SMD = 2.82, 95% CI: -0.02 to 5.65, *p* = 0.05). Because the 95% CI crossed zero, this result was interpreted cautiously and was not described as statistically significant. Data for Osteocalcin (OC) were pooled from five studies (SMD = -0.08, 95% CI: -3.20 to 3.03, *p* = 0.96). Additionally, five studies reported on serum Calcium (Ca) levels (SMD = 1.01, 95% CI: -0.28 to 2.30, *p* = 0.13), and five studies analyzed serum Phosphorus (P) (SMD = 0.23, 95% CI: -0.19 to 0.65, *p* = 0.29).

### 3.7 Cytokines

**Figure 8:**
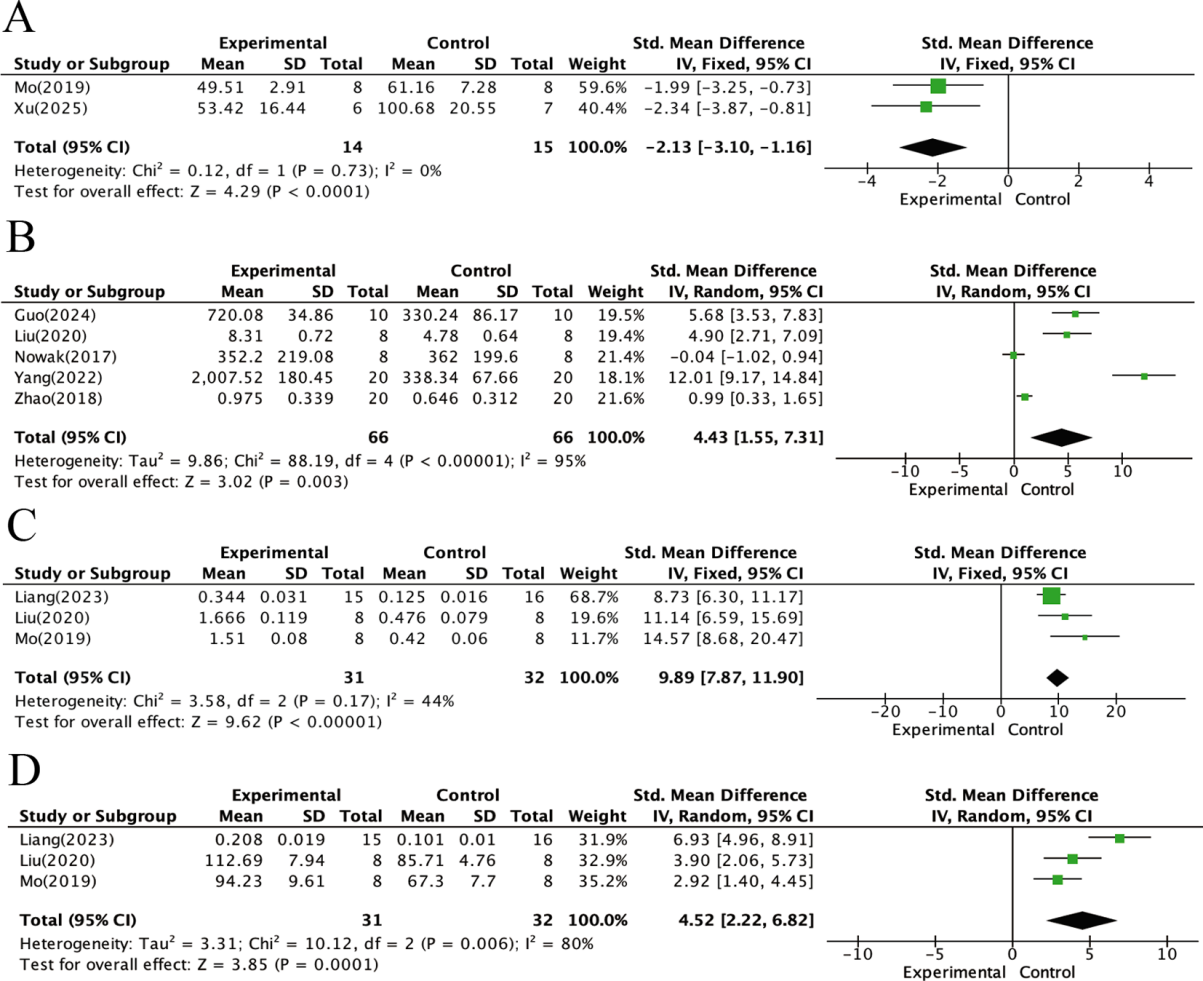
Forest plot of cytokines and growth factors. **(A)** IL-6; **(B)** OPG; **(C)** IGF; **(D)** TGF-β. Effect size: SMD. Negative values for IL-6 indicate lower inflammatory cytokine levels in the *Semen Cuscutae* group and favor *Semen Cuscutae*; positive values for OPG, IGF, and TGF-β indicate higher levels in the *Semen Cuscutae* group and favor *Semen Cuscutae*.

**Figure 8** summarizes the meta-analysis results regarding changes in cytokine levels following *Semen Cuscutae* intervention in osteoporotic animal models. Two studies indicated that *Semen Cuscutae* significantly reduced the levels of IL-6 (SMD = -2.13, 95% CI: -3.10 to -1.16, *p* < 0.001). Conversely, five studies reported a significant increase in OPG levels (SMD = 4.43, 95% CI: 1.55 to 7.31, *p* = 0.003). Similarly, significant elevations were observed for IGF (3 studies; SMD = 9.89, 95% CI: 7.87 to 11.90, *p* < 0.001) and TGF-β (3 studies; SMD = 4.52, 95% CI: 2.22 to 6.82, *p* < 0.001).

### 3.8 Sensitivity analysis results

**Figure 9:**
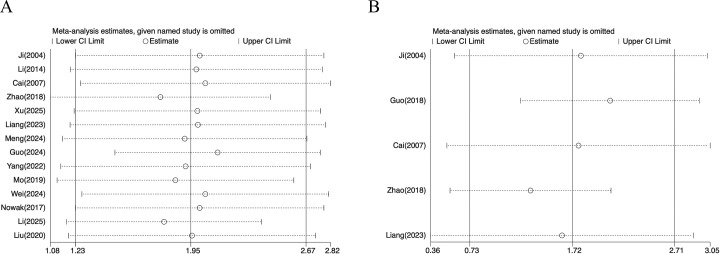
Sensitivity analysis of bone mineral density. **(A)** Femur BMD; **(B)** Lumbar spine BMD.

Sensitivity analysis was performed using the “leave-one-out” approach, as illustrated in **Figure 9**. Following the sequential exclusion of each individual study, the pooled effect sizes for both femur and lumbar spine BMD consistently remained within the upper and lower limits of the 95% CI. Furthermore, all point estimates and their corresponding 95% CIs did not cross the line of no effect, indicating that the direction of the overall effect remained unchanged and confirming the robustness of the conclusions. No single study was observed to exert a disproportionate influence on the pooled effect size, demonstrating the high stability of the meta-analysis results.

### 3.9 Machine learning results

**Figure 10:**
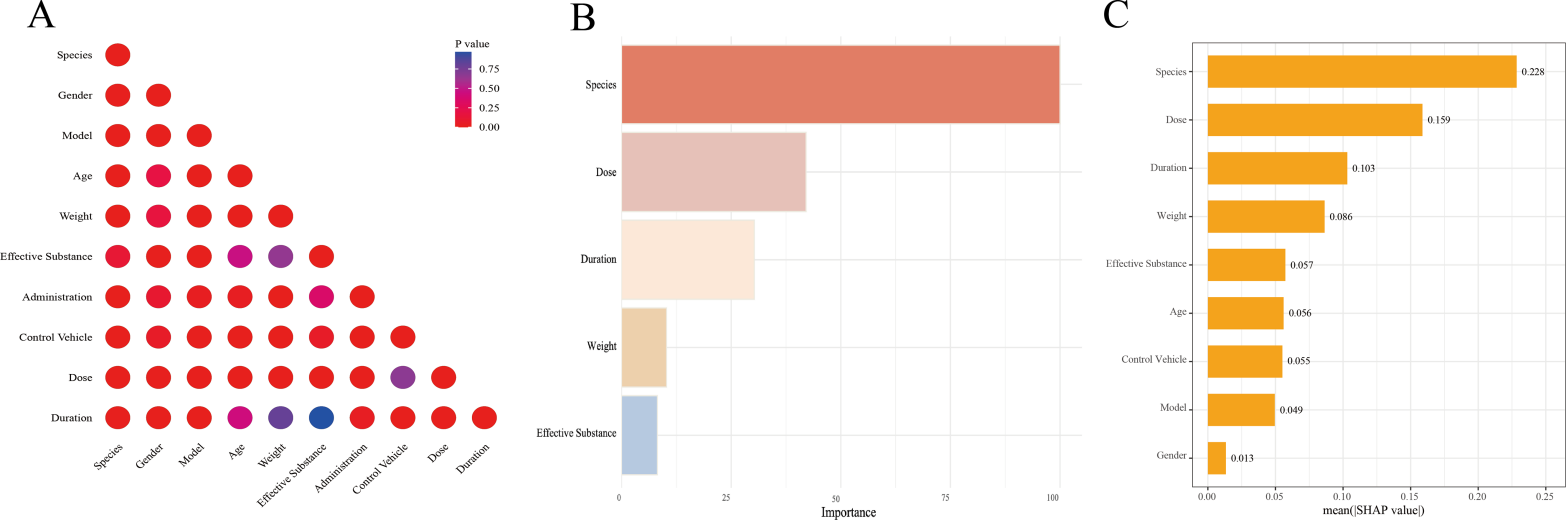
Exploratory machine learning analysis ranking experimental variables potentially associated with *Semen Cuscutae* efficacy. **(A)** Pearson correlation heatmap providing a preliminary visualization of pairwise associations among ten encoded experimental variables. **(B)** Feature importance ranking derived from the eXtreme Gradient Boosting (XGBoost) model. The length of the bar represents the relative importance of each feature in the exploratory model. **(C)** SHAP (SHapley Additive exPlanations) summary plot illustrating the mean absolute SHAP value of each feature. The x-axis represents the average contribution of each variable to the model output. Species, Dose, and Duration were ranked as the top three variables potentially associated with treatment efficacy in the current dataset.

Based on the constructed structured dataset comprising 10 key experimental variables, interactions between features were initially assessed using Pearson correlation analysis. As visualized in the correlation heatmap (**Figure 10A**), statistical associations between variables were examined, and this analysis was used as a preliminary visualization of pairwise associations among encoded variables rather than as definitive evidence excluding multicollinearity. Subsequently, the XGBoost algorithm, integrated with the SHAP interpretability framework, was utilized as an exploratory tool to rank variables potentially associated with the efficacy of Semen Cuscutae. Using the 70/30 train-test split, the internal test-set performance of the exploratory XGBoost model was R² = -0.365, RMSE = 5.383, and MAE = 2.973. Given the limited number of included studies and the small size of the test set, these metrics indicate limited predictive performance and should be interpreted only as descriptive indicators of internal model behavior rather than evidence of external predictive validity. Feature importance analysis (**Figure 10B**) preliminarily identified Species, Dose, and Duration as the top-ranking variables associated with model output. Quantitative analysis of SHAP values (**Figure 10C**) further supported this ranking. Specifically, Species exhibited the highest contribution to the model output, with a mean absolute SHAP value of 0.228, suggesting that the choice of animal species may be an important factor associated with the observed treatment effect. This was followed by Dose (mean |SHAP value| = 0.159) and Duration (0.103), indicating that intervention regimen-related factors may contribute to heterogeneity in therapeutic efficacy. Furthermore, Weight (0.086), Active component (0.057), and Age (0.056) demonstrated moderate contributions, whereas Gender exhibited the lowest SHAP value (0.013). Overall, this ML-derived ranking should be interpreted as exploratory and requires validation in larger, standardized preclinical datasets.

## 4 Discussion

By integrating data from 15 eligible preclinical studies, this study represents the first systematic evaluation using a combined strategy of meta-analysis and machine learning (specifically, the XGBoost algorithm). We systematically evaluated the efficacy of *Semen Cuscutae* in treating osteoporosis (OP) and explored potential experimental factors associated with treatment efficacy. From an evidence-based perspective, our findings suggest that *Semen Cuscutae* may enhance Bone Mineral Density (BMD) and improve bone micro-architecture, while the SHAP interpretability model provided an exploratory ranking of efficacy-associated variables. Consequently, this work provides preliminary evidence to support the translational evaluation of natural therapeutic agents from empirical practice toward precision medicine (22, 41).

Our meta-analysis demonstrates that *Semen Cuscutae* intervention significantly increases BMD in the femur and lumbar spine, and improves trabecular micro-architecture, characterized by increased BV/TV and Tb.Th, and decreased Tb.Sp. The observed enhancement in biomechanical performance (specifically, ultimate stress) further corroborates a substantial improvement in bone quality. Mechanistically, this osteoprotective effect is attributed to the remodeling of the “osteoimmune microenvironment.”.

Firstly, regarding bone remodeling homeostasis, the increasing trend in serum ALP and the significant upregulation of OPG levels (SMD = 4.43) suggest that Semen Cuscutae may promote osteoblast differentiation and bone formation. Crucially, our analysis highlights the pivotal role of *Semen Cuscutae* in anti-inflammation and osteoimmune modulation. The meta-analysis demonstrated that *Semen Cuscutae* intervention significantly reduced the levels of the pro-inflammatory cytokine IL-6 (SMD = -2.13). In the pathogenesis of postmenopausal osteoporosis, estrogen withdrawal typically precipitates chronic low-grade inflammation (often termed “Inflamm-aging”), leading to the accumulation of pro-inflammatory cytokines, such as IL-6 and TNF-α, within the bone marrow microenvironment. These cytokines act as potent osteoclast activators; by upregulating RANKL expression and activating the NF-κB signaling pathway, they induce excessive differentiation of osteoclast precursors, thereby establishing a vicious cycle of “inflammation-induced bone loss” (42, 43). The significant reduction in IL-6 observed in this study aligns closely with the decreasing trend of bone resorption markers (TRACP and CTX), suggesting that *Semen Cuscutae* may exert its effects by blocking the “IL-6/RANKL/NF-κB” axis, effectively disrupting the inflammation-driven bone resorption cascade.

Furthermore, the amelioration of the inflammatory microenvironment creates a favorable milieu for bone repair (44, 45). Our data indicate that, concomitant with the suppression of inflammation, the expression levels of pro-reparative factors IGF (SMD = 9.89) and TGF-β (SMD = 4.52) were significantly restored. TGF-β serves not only as a pivotal chemotactic factor for osteoblast recruitment but also synergistically attenuates hyperactive immune responses (46). This suggests that *Semen Cuscutae* does not solely inhibit bone resorption; rather, it re-establishes bone metabolic homeostasis through a coupled “anti-inflammatory and pro-repair” mechanism.

Notably, despite significant improvements in BMD and primary bone metabolic indices, no statistically significant changes were observed in serum Osteocalcin (OC), Calcium (Ca), and Phosphorus (P) levels. Rather than implying therapeutic inefficacy, these findings likely reflect the body’s intricate compensatory mechanisms and the temporal discrepancies associated with bone turnover markers (47). First, serum Ca and P levels are subject to rigid homeostatic regulation by parathyroid hormone (PTH) and renal function. Even in severe osteoporotic states, the body prioritizes the maintenance of physiological serum calcium levels via “skeletal calcium mobilization,” thereby rendering these systemic markers insufficiently sensitive to local pathological changes in the skeleton (48). Second, the lack of significant change in the osteogenic marker OC (*p* = 0.96) may be attributed to the “coupling effect” of bone remodeling. As *Semen Cuscutae* potently inhibits bone resorption (evidenced by the substantial decline in TRACP/CTX), the high-turnover metabolic state is suppressed. This suppression may lead to a secondary reduction in the overall bone turnover rate, effectively masking the net increase in the late-stage osteogenic marker, OC (49). In contrast, the increasing trend in ALP, an early-stage marker, may more sensitively reflect the initial activation signals of osteoblasts (50).

Several pooled SMDs in this study were large, particularly for bone resorption markers and cytokine-related outcomes. These large standardized effects should be interpreted cautiously. First, SMD is a scale-free measure that is strongly influenced by the within-study standard deviation; therefore, small standard deviations in controlled animal experiments can produce large standardized effect estimates even when the absolute biological difference is moderate. Second, many included studies had relatively small sample sizes, which may increase random error and contribute to unstable effect-size estimates. Third, differences in assay platforms, measurement units, animal models, intervention regimens, and tissue or serum sampling procedures may further increase heterogeneity and affect standardized estimates. Finally, incomplete reporting of allocation concealment and blinding may increase the risk of effect overestimation. Therefore, the large SMDs observed in this preclinical meta-analysis should not be directly interpreted as proportionally large clinical effects, but rather as indicators of the direction and relative magnitude of treatment-associated changes within the available animal evidence.

While traditional subgroup analyses are often limited to assessing univariate linear relationships, this study incorporated the XGBoost model and SHAP analysis as an exploratory complement to conventional meta-analytic methods. This approach was used to generate a data-driven ranking of experimental variables potentially associated with treatment efficacy, rather than to establish a definitive prediction model (51). A notable finding from the SHAP ranking (**Figure 10C**) is that “Species” serves not only as a major source of heterogeneity but also as the primary predictor of therapeutic efficacy (SHAP value = 0.228). Specifically, rat models exhibited a superior therapeutic response compared to mice. This discrepancy may be attributable to variations in bone metabolic rates and pharmacokinetic (PK) profiles between these rodent species (52, 53). Consequently, this finding suggests that interspecies differences should be considered carefully in future preclinical design and translational dose extrapolation, rather than relying on simplistic dose conversions.

Furthermore, Dose and Duration ranked second and third, respectively, in terms of SHAP-based feature importance. This finding suggests that the administration regimen may be an important contributor to heterogeneity in the observed effects of *Semen Cuscutae* (54, 55). In contrast, Gender showed the lowest SHAP value. This result may stem from the predominance of ovariectomized models among the included studies, which led to a skew toward female samples in the dataset and may have masked the potential influence of sex-related biological differences. Therefore, this finding should not be interpreted as evidence that gender has no biological relevance, but rather as an exploratory observation derived from the currently available dataset (15).

Although the sensitivity analyses supported the stability of the meta-analytic findings, several limitations must be acknowledged. First, the exploratory ML analysis should be interpreted with caution. The number of included studies was limited, and the feature-to-sample ratio was relatively high, which may increase the risk of overfitting and reduce the reproducibility of model-derived rankings. In addition, although a 70/30 train-test split was used to follow the original analytical framework, the test set contained only five study-level records, making the performance estimates unstable and sensitive to individual studies. The internal test-set performance of the model was R² = -0.365, RMSE = 5.383, and MAE = 2.973, indicating limited predictive performance. Therefore, the XGBoost-SHAP analysis in this study should not be regarded as a definitive predictive model, but rather as a complementary exploratory tool for hypothesis generation and feature prioritization. Larger datasets with standardized experimental reporting are required to validate the stability, reproducibility, and external generalizability of these ML-derived findings. Second, the methodological quality of the included studies should be interpreted cautiously. Although all included studies reported adequate sequence generation and were free from incomplete outcome data and selective reporting, several key domains of the SYRCLE risk-of-bias tool were insufficiently reported. In particular, allocation concealment was not reported in several studies, and none of the included studies reported caregiver blinding, randomized outcome assessment, or blinded outcome assessment. These deficiencies may introduce selection bias, performance bias, and detection bias, respectively, and may therefore lead to overestimation of the intervention effects. Consequently, although the pooled results suggest a potential osteoprotective effect of *Semen Cuscutae*, the overall credibility of the evidence is constrained by these methodological limitations. Future preclinical studies should adopt and clearly report rigorous randomization procedures, allocation concealment, blinded intervention administration, randomized outcome assessment, and blinded outcome assessment to improve the reliability and translational value of the evidence. In addition, several outcomes showed very large SMDs, which may be partly attributable to small sample sizes, narrow within-study variability, and methodological limitations in the included animal studies. These large standardized effects should therefore be interpreted cautiously and should not be directly extrapolated to clinical effect sizes. Third, the included studies predominantly utilized rodent models, including rats and mice, with a notable absence of data from non-human primates. This constraint limits, to a certain extent, the direct extrapolation of these findings to clinical settings. Fourth, due to the lack of detailed reporting on specific compound concentrations in some primary studies, we were precluded from performing a more granular structure–activity relationship analysis of the active constituents. In addition, although *Semen Cuscutae* is traditionally used as a whole herbal medicine from the perspective of traditional Chinese medicine and ethnopharmacology, the pharmacological diversity of the included *Semen Cuscutae*-derived preparations should still be considered when interpreting the pooled estimates, because several preparation categories were represented by only a limited number of studies.

In conclusion, *Semen Cuscuta*e showed anti-osteoporotic potential through the bidirectional regulation of bone metabolism and the suppression of inflammatory responses. The exploratory machine learning analysis suggests that treatment duration and interspecies dose scaling may warrant consideration in future preclinical and translational research. This study provides preliminary preclinical evidence for the evidence-based evaluation of natural medicines against osteoporosis and demonstrates the potential utility of exploratory artificial intelligence-based approaches in integrating complex medical evidence.

## Data Availability

The original contributions presented in the study are included in the article/**Supplementary Material**. Further inquiries can be directed to the corresponding authors.
