# Supplementary material for: Unveiling the efficacy predictors and potential mechanisms of Semen Cuscutae against osteoporosis via machine learning and meta-analysis: a preclinical study
Source: Front Immunol. 2026 Jun 10;17:1766283. doi: 10.3389/fimmu.2026.1766283 (PMC13290541; doi:10.3389/fimmu.2026.1766283)
Supplement: Supplementary file 1 [file Supplementaryfile1.docx]

**Pubmed**

("Cuscuta"[Mesh] OR "Cuscuta"[tiab] OR "Cuscuta chinensis"[tiab] OR "Semen cuscutae"[tiab] OR "dodder seeds"[tiab] OR "Tu Si Zi"[tiab]) AND ("Rats"[Mesh] OR "Mice"[Mesh] OR "Rodentia"[Mesh] OR "Animals, Laboratory"[Mesh] OR rat[tiab] OR rats[tiab] OR mouse[tiab] OR mice[tiab] OR rodent[tiab] OR "animal experiment*"[tiab] OR "ovariectomized rat"[tiab] OR "ovariectomized mouse"[tiab]) AND ("Osteoporosis"[Mesh] OR osteoporosis[tiab] OR "bone loss"[tiab] OR "bone density"[tiab])

**Web of science**

TS=(Cuscuta OR "Cuscuta chinensis" OR "Semen cuscutae" OR "dodder seeds" OR "Tu Si Zi") AND TS=(Rat OR Rats OR Mouse OR Mice OR Rodent* OR "Laboratory Animal*" OR "ovariectomized rat" OR "ovariectomized mouse") AND TS=(Osteoporosis OR "bone loss" OR "bone density")

**Embase**

('Cuscuta'/de OR 'Cuscuta':ti,ab OR 'Cuscuta chinensis':ti,ab OR 'Semen cuscutae':ti,ab OR 'dodder seeds':ti,ab OR 'Tu Si Zi':ti,ab) AND ('Rat'/de OR 'Mouse'/de OR 'Rodent'/de OR 'Laboratory Animal'/de OR rat:ti,ab OR rats:ti,ab OR mouse:ti,ab OR mice:ti,ab OR rodent:ti,ab OR 'animal experiment*':ti,ab OR 'ovariectomized rat':ti,ab OR 'ovariectomized mouse':ti,ab) AND ('Osteoporosis'/de OR osteoporosis:ti,ab OR 'bone loss':ti,ab OR 'bone density':ti,ab)

**Foreign Medical Literature Retrieval Service**

(Cuscuta[TIAB] OR "Cuscuta chinensis"[TIAB] OR "Semen cuscutae"[TIAB] OR "dodder seeds"[TIAB] OR "Tu Si Zi"[TIAB]) AND (rat[TIAB] OR rats[TIAB] OR mouse[TIAB] OR mice[TIAB] OR rodent*[TIAB] OR "ovariectomized rat"[TIAB] OR "ovariectomized mouse"[TIAB]) AND (osteoporosis[TIAB] OR "bone loss"[TIAB] OR "bone density"[TIAB])

**Scopus**

TITLE-ABS-KEY ( "Cuscuta" OR "Cuscuta chinensis" OR "Semen cuscutae" OR "dodder seeds" OR "Tu Si Zi" ) AND TITLE-ABS-KEY ( rat OR rats OR mouse OR mice OR rodent* OR "ovariectomized rat" OR "ovariectomized mouse" ) AND TITLE-ABS-KEY ( osteoporosis OR "bone loss" OR "bone density" )
